# Supplementary material for: Fungal diversity in shade-coffee plantations in Soconusco, Mexico
Source: PeerJ. 2022 Jun 29;10:e13610. doi: 10.7717/peerj.13610 (PMC9250310; doi:10.7717/peerj.13610)
Supplement: Supplemental Information 1 [file peerj-10-13610-s001.pdf]

Supplementary material – File S1

Manuscript submitted to PeerJ: “Fungal diversity in shade-coffee plantations in Soconusco, Mexico”

Eugenia Zarza[1,2], Alejandra López-Pastrana[3], Anne Damon[3*], Karina Guillén-Navarro[1+], Luz Verónica García-Fajardo[1].

[1] Departamento de Ciencias de la Sustentabilidad, El Colegio de la Frontera Sur (ECOSUR), Tapachula, Chiapas, Mexico; [2] CONACYT, Ciudad de México, Mexico; [3] Departamento de Conservación de la Biodiversidad, El Colegio de la Frontera Sur (ECOSUR), Tapachula, Chiapas, Mexico.

Corresponding authors:

***Anne Damon** adamon@ecosur.mx

+ **Karina Guillén-Navarro:** kguillen@ecosur.mx

Data analyzed by:

EZ eugenia.zarza@ecosur.mx

Table S1. Bark sample ID, origin, number of raw sequences, number of filtered and denoised sequences for all taxa and Fungi. Samples were obtained from four microsites on coffee bushes and shade trees in two shade-coffee plantations in Soconusco, Chiapas, Mexico.

SampleID	Plant type	Microsite	Raw sequences	All taxa	Fungi
BOP1	coffee	Twig	214,165	106,772	38,975
BOP6	coffee	Branch	164,729	78,131	30,054
BOP11	coffee	Trunk high	193,103	103,311	89,072
BOP16	coffee	Trunk low	256,570	131,900	88,364
BOP22	coffee	Twig	183,399	81,562	17,974
BOP27	coffee	Branch	171,632	84,479	50,748
BOP32	coffee	Trunk high	211,255	102,650	87,843
BOP37	coffee	Trunk low	192,074	97,132	71,492
BOP43	tree	Twig	182,587	93,745	18,624
BOP48	tree	Branch	170,527	94,852	34,055
BOP53	tree	Trunk high	195,042	103,919	59,785
BOP58	tree	Trunk low	205,733	111,434	35,800
BOP66	coffee	Twig	186,238	96,382	94,364
BOP69	coffee	Branch	175,926	86,234	48,725
BOP74	coffee	Trunk high	204,217	123,395	112,248
BOP79	coffee	Trunk low	179,709	93,804	79,128
BOP87	coffee	Twig	202,014	99,874	39,368
BOP90	coffee	Branch	220,739	117,218	94,007
BOP95	coffee	Trunk high	189,933	86,382	25,499
BOP100	coffee	Trunk low	207,257	111,815	82,300
BOP109	tree	Twig	170,471	57,023	20,002
BOP111	tree	Branch	180,391	60,856	24,825
BOP116	tree	Trunk high	192,161	77,240	55,215
BOP121	tree	Trunk low	162,061	48,608	16,387
HOP1	coffee	Twig	195,562	82,646	9,260
HOP6	coffee	Branch	185,895	88,435	56,024
HOP11	coffee	Trunk high	222,286	120,780	67,214
HOP16	coffee	Trunk low	223,224	140,012	128,158
HOP23	coffee	Twig	234,696	119,920	117,590
HOP27	coffee	Branch	142,544	51,828	32,812
HOP32	coffee	Trunk high	195,345	89,824	18,607
HOP37	coffee	Trunk low	184,532	91,906	45,672
HOP47	tree	Twig	207,448	71,385	35,766
HOP48	tree	Branch	225,203	79,503	45,926
HOP53	tree	Trunk high	210,404	87,201	79,237
HOP58	tree	Trunk low	186,010	98,152	80,549
HOP64	coffee	Twig	191,824	90,763	37,672

HOP69	coffee	Branch	177,532	84,093	57,604
HOP74	coffee	Trunk high	185,834	77,152	59,008
HOP79	coffee	Trunk low	185,130	90,359	61,595
HOP85	coffee	Twig	187,831	59,789	12,478
HOP90	coffee	Branch	220,986	123,865	106,949
HOP95	coffee	Trunk high	212,014	87,394	36,610
HOP100	coffee	Trunk low	249,566	92,746	20,501
HOP106	tree	Twig	192,019	43,161	15,722
HOP111	tree	Branch	191,657	55,069	31,743
HOP118	tree	Trunk high	179,614	34,492	2,884
HOP121	tree	Trunk low	144,026	9,357	5,269

Prefix BOP = Benito Juárez El Plan; Prefix HOP = Los Hermanitos

Table S2. Taxonomic classification of top ten most abundant taxa, obtained with the BLAST tool implemented in QIIME2 view, detected in bark samples of coffee bushes and shade trees in two coffee plantations in Soconusco region, Chiapas, Mexico.

Taxon	Classification (Kingdom; Family)	Sequence counts	Number of samples	Identity (%)	Bit score
Coffea arabica	Viridiplantae; Rubiaceae	801,210	27	100	435
Coffea canephora	Viridiplantae; Rubiaceae	281,064	8	100	435
Viburnum hartwegii	Viridiplantae; Adoxaceae	250,446	6	99.58	429
Frangula purshiana	Viridiplantae; Rhamnaceae	124,286	4	98.75	420
Trogia aff. *furcata*	Fungi; Tricholomataceae	122,201	2	97.08	398
Xanthoparmelia aff. *hottentota*	Fungi; Parmeliaceae	78,020	4	75.53	118
Phyllopsora confusa	Fungi; Ramalinaceae	60,983	2	95.79	415
Inga alata	Viridiplantae; Fabaceae	56,409	4	99.17	426
Uncultured Fungi	Fungi	53,804	3	79.02	176
Guarea glabra	Viridiplantae; Meliaceae	49,160	3	98.33	413

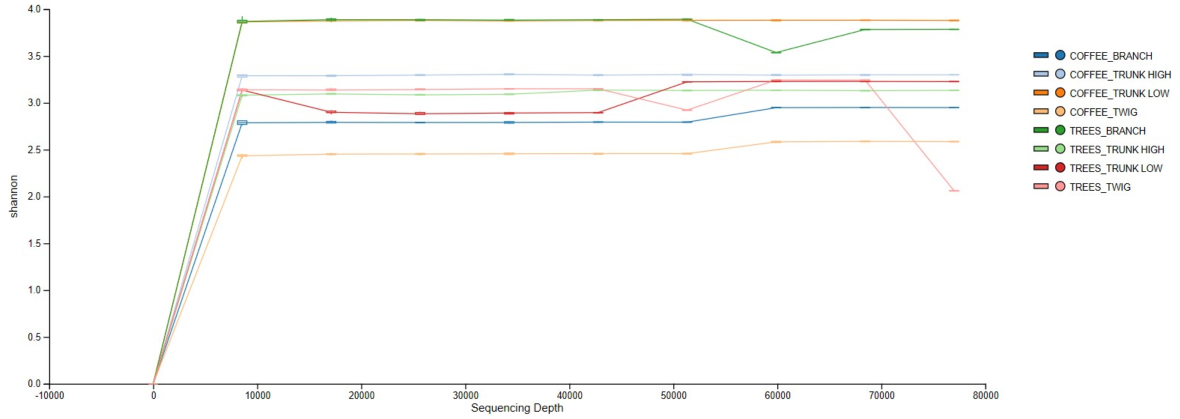

Figure S1. Alpha rarefaction plot, calculated with QIIME2, for all bark samples included in each treatment and grouped according to plant type (coffee bush, shade tree) and plant microsite (branch, trunk high, trunk low, twig).

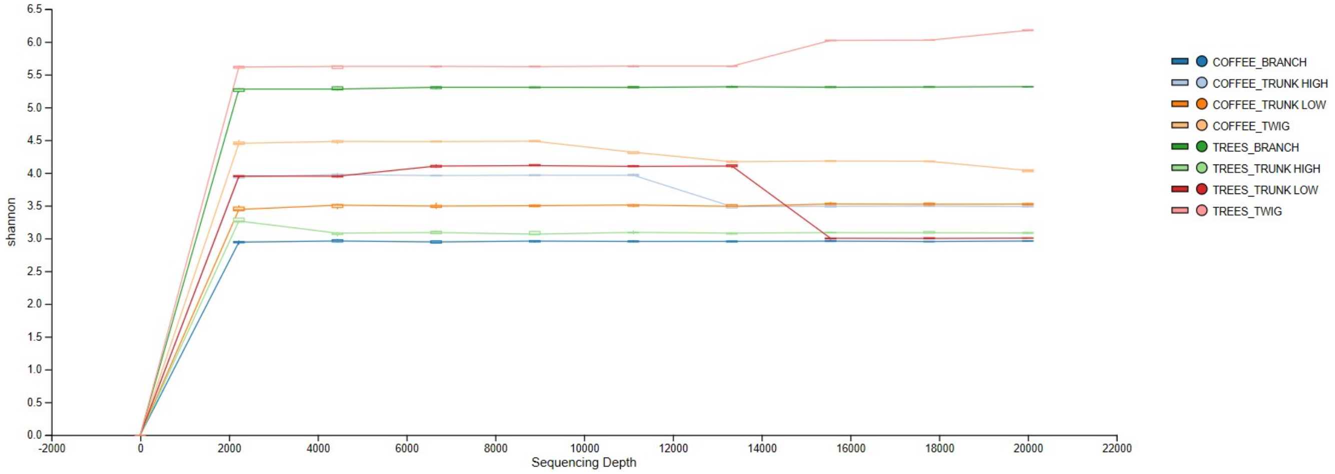

Figure S2. Alpha rarefaction plot, calculated with QIIME2, for bark samples grouped according to plant type (coffee bush, shade tree) and plant microsite (branch, trunk high, trunk low, twig), and including only sequences classified as Fungi.

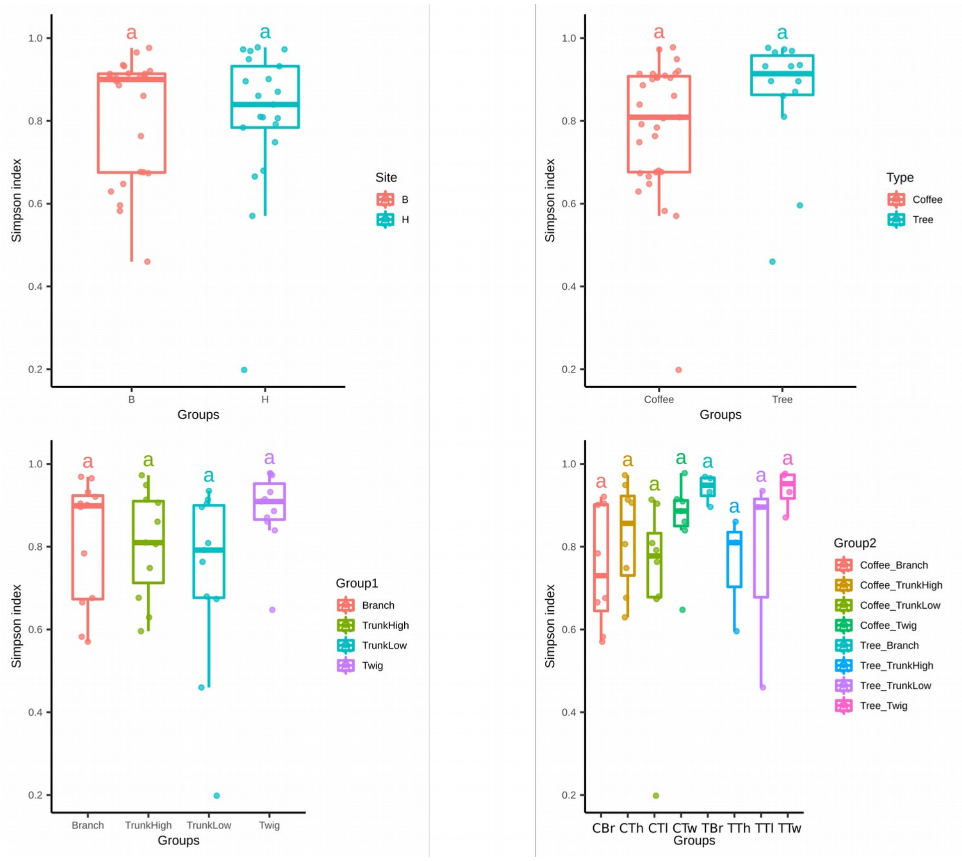
Simpson index
Groups
Site
B
H
Type
Coffee
Tree
Group1
Branch
TrunkHigh
TrunkLow
Twig
Group2
Coffee_Branch
Coffee_TrunkHigh
Coffee_TrunkLow
Coffee_Twig
Tree_Branch
Tree_TrunkHigh
Tree_TrunkLow
Tree_Twig
CBr CTh CTl CTw TBr TTh TTl TTw
a

a

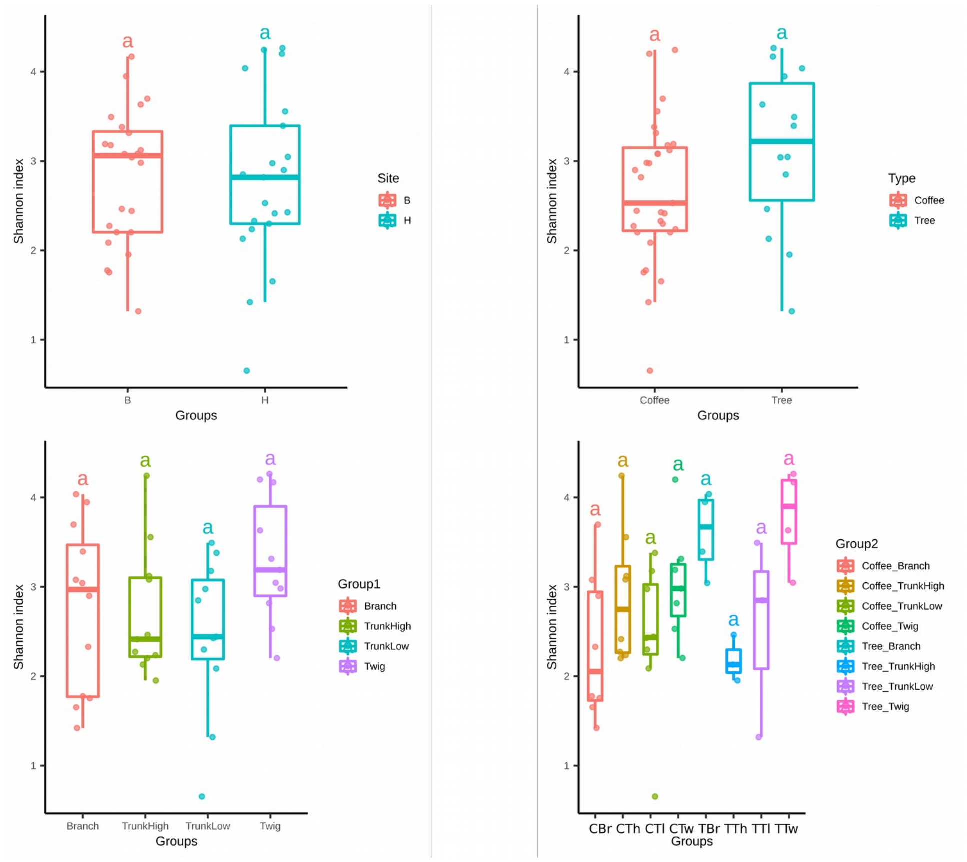

b

Figure S3. Boxplots of Simpson (a) and Shannon (b) indeces with rarefraction at 12,408 reads (lowest number of reads per sample after removing reads with less than 10,000 reads), according to collection site (B: Benito Juárez El Plan, H: Hermanitos), plant type, sampled plant part and microsite (CTw: coffe twig; CTh: coffee trunk high; CTl: coffee trunk low; CBr: coffee branch; TTw: tree twig; TTh: tree trunk high; TTl: tree trunk low; TBr: tree branch)

a

method name	PERMANOVA
test statistic name	pseudo-F
sample size	38
number of groups	2
test statistic	2.51249
p-value	0.004
q-value	0.004
number of permutations	999

b c

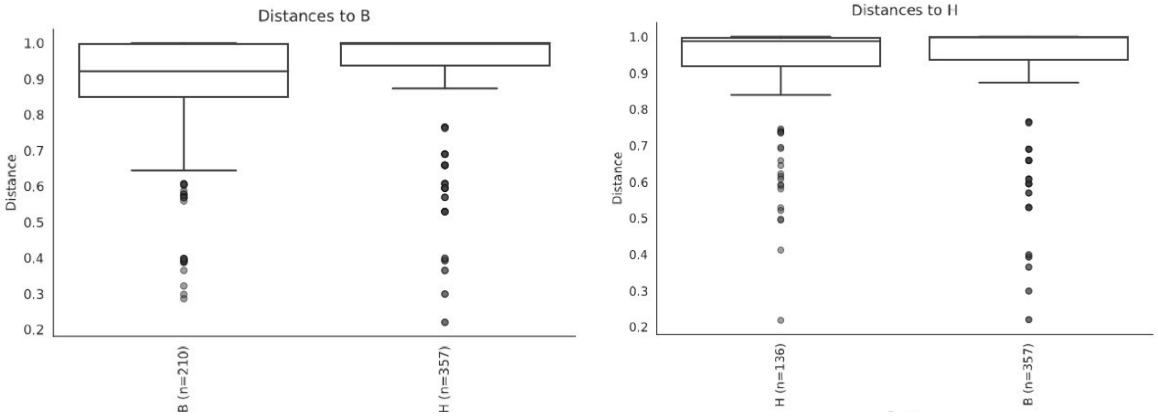

Figure S4. Group significance associations per site using Bray-Curtis distance, calculated with QIIME2, based on the dataset including all taxa detected in bark samples of coffee bushes and shade trees in two coffee plantations in Soconusco region, Chiapas, Mexico. a) Details for PERMANOVA test; b) Distances to sample group ‘site B’; c) Distances to sample group ‘site H’. B= Benito Juárez El Plan; H = Los Hermanitos.

a

method name	PERMANOVA
**test statistic name**	pseudo-F
**sample size**	38
**number of groups**	2
**test statistic**	4.05991
**p-value**	0.001
**q-value**	0.001
**number of permutations**	999

b

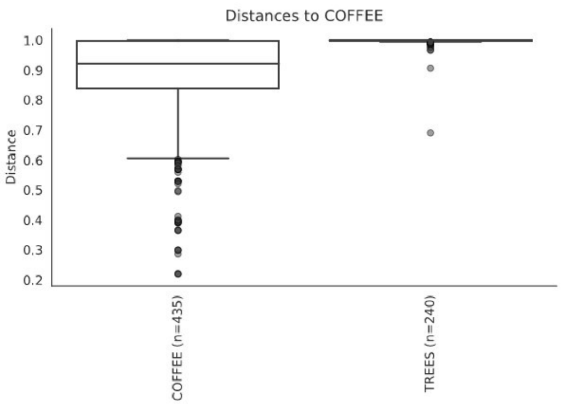

c

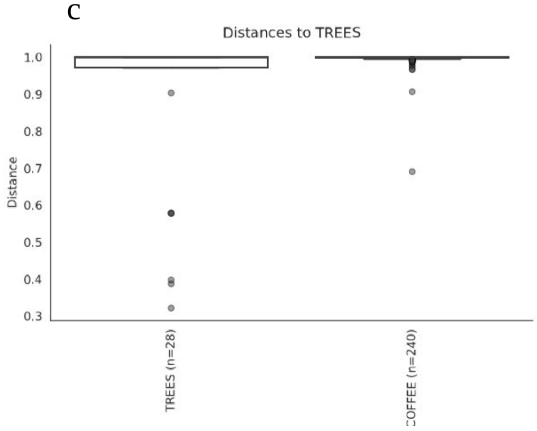

Figure S5. Group significance associations per plant type using Bray-Curtis distance based on the dataset including all taxa detected in bark samples of coffee bushes and shade trees in two coffee plantations in Soconusco region, Chiapas, Mexico. a) Details for PERMANOVA test; b) Distances to sample group 'coffee bushes'; c) Distances to sample group 'shade trees'. Calculated with QIIME2.

a

method name	PERMANOVA
**test statistic name**	pseudo-F
**sample size**	37
**number of groups**	2
**test statistic**	1.64432
**p-value**	0.001
**q-value**	0.001
**number of permutations**	999

b c

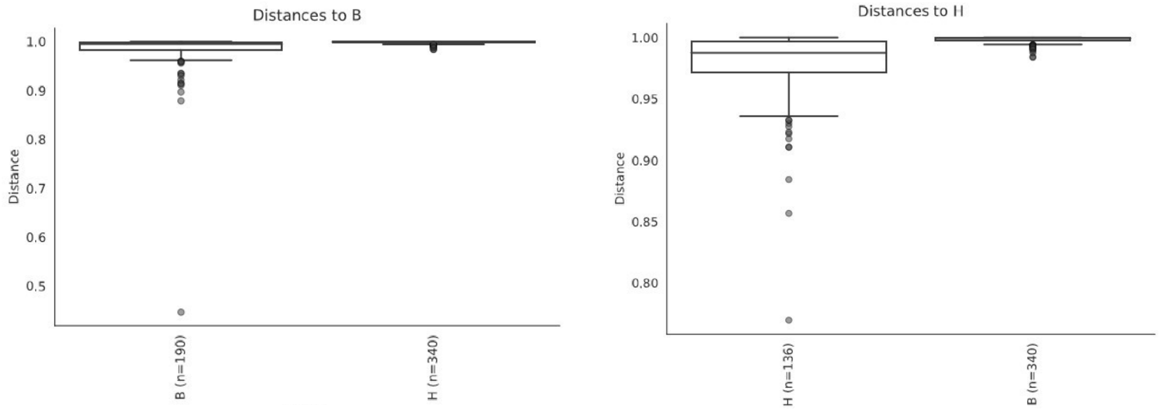

Figure S6. Group significance associations per site using Bray-Curtis distance based on only Fungi detected in bark samples of coffee bushes and shade trees in two coffee plantations in Soconusco region, Chiapas, Mexico. a) Details for PERMANOVA test; b) Distances to sample group 'site B'; c) Distances to sample group 'site H'. B= Benito Juárez El Plan; H = Los Hermanitos. Calculated with QIIME2.
